# Leveraging tobacco for a low-carbon biorefinery

**DOI:** 10.3389/fpls.2025.1662412

**Published:** 2025-12-16

**Authors:** Quanyu Yin, Zhengkang Zhu, Yujia Chen, Mengquan Yang

**Affiliations:** National Tobacco Cultivation, Physiology and Biochemistry Research Center, Flavors and Fragrance Engineering and Technology Research Center of Henan Province, College of Tobacco Science, Henan Agricultural University, Zhengzhou, Henan, China

**Keywords:** tobacco biorefinery, low-carbon, autoclave hydrolysis, life cycle assessment, policy alignment

## Abstract

Tobacco has historically served as a high-value cash crop within the cigarette industry. However, increasing public health concerns and climate change mitigation objectives are driving a reassessment of its applications. Given its substantial biomass yield and well-established agricultural infrastructure, tobacco is being investigated as a potential renewable energy crop. Recent studies indicate that tobacco leaves can be effectively converted into fermentable substrates through a straightforward autoclave-based hydrolysis process, yielding a nutrient-rich solution suitable for biofuel fermentation and offering a favorable environmental profile. Building on these insights, this perspective evaluates the potential of utilizing tobacco as a feedstock in low-carbon biorefineries. We conduct a critical analysis of recent advancements in biomass conversion, life cycle assessment, utilization of agricultural residues, microbial compatibility, agronomic and genetic enhancements, land-use implications, and policy frameworks. Key challenges and future research directions are identified to facilitate the transformation of tobacco from a contentious crop into a source of sustainable fuels and bioproducts.

## Introduction

Tobacco (*Nicotiana tabacum* L.) has historically been cultivated globally as a profitable crop for the production of cigarettes and other smoking products (Feng et al., 2023; Lian et al., 2023). Nevertheless, the decreasing demand for tobacco products, driven by public health initiatives and the critical need to mitigate greenhouse gas emissions, has generated interest in exploring alternative applications for this extensively researched plant (Ti et al., 2024; Abdellah et al., 2025; Liu et al., 2025; Manthos and Tsigkou, 2025). The high biomass productivity of tobacco, coupled with a comprehensive agronomic knowledge base and established farmer networks, positions it as a promising feedstock candidate for biofuels and bioproducts (Barla and Kumar, 2019; Reynolds et al., 2022; Liu et al., 2025; Ramontja et al., 2025). Recent studies have demonstrated that tobacco leaves can be directly converted into a fermentable medium with minimal processing: Wang et al. (2024) reported achieving over 65% solubilization of tobacco leaf biomass through simple autoclaving in water, resulting in a nutrient-rich hydrolysate capable of supporting microbial growth without the need for expensive pretreatments. The observed efficiency can be attributed to the composition of tobacco leaves, which are rich in water-soluble carbohydrates and nitrogen while containing relatively low levels of lignin. This composition simplifies processing and reduces carbon intensity. Furthermore, the bioethanol production process derived from tobacco demonstrates a markedly improved environmental footprint compared to traditional biofuels (Wang et al., 2024).

**Figure 1:**
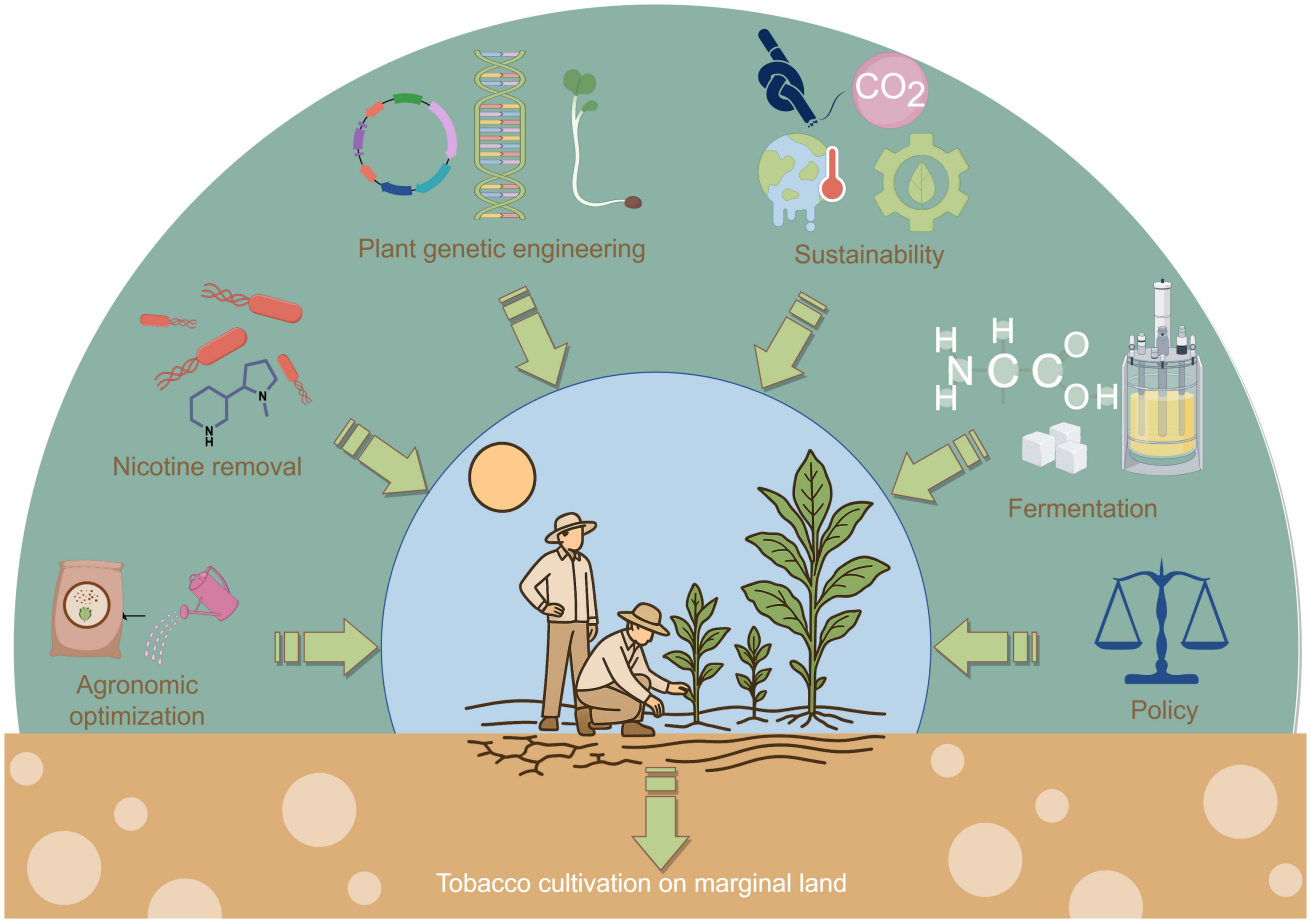
Conceptual overview of a tobacco-based low-carbon biorefinery. The schematic illustrates the pathway of converting tobacco biomass into biofuel: tobacco leaves are subjected to a simple autoclave hydrolysis to produce a fermentable, nutrient-rich broth, which is then used for microbial ethanol fermentation. It also emphasizes cultivation on marginal lands and supportive policy frameworks to enable a sustainable tobacco biorefinery. (Diagram created with Figdraw.).

However, transitioning tobacco from a crop used for smoking to a bioenergy feedstock raises several critical questions. What are the environmental trade-offs associated with large-scale tobacco biofuel production on marginal lands? How can nicotine toxicity be effectively managed to ensure successful microbial fermentation? Additionally, what roles do socioeconomic and policy factors play in facilitating or hindering this transition? This review addresses these questions by synthesizing current knowledge and identifying future research needs. In the subsequent sections, we explore recent advancements and challenges in biomass conversion, life cycle assessment, valorization of tobacco residues, microbial compatibility, agronomic and genetic optimization, sustainable land use, techno-economic feasibility, and supportive policy measures for the development of low-carbon tobacco-based biofuels (**Figure 1**).

## Biomass conversion

Traditional lignocellulosic biorefineries typically necessitate energy-intensive chemical pretreatments and the use of enzyme cocktails to deconstruct complex plant polymers into fermentable sugars (Saini et al., 2022; Ding et al., 2024). However, Wang et al. (2024) have addressed these challenges by implementing a straightforward autoclave-based hydrolysis method on tobacco leaves (Wang et al., 2024). This process involves sterilizing fresh tobacco leaves in water under moderate temperature and pressure conditions, resulting in a hydrolysate where over 65% of the leaf biomass is solubilized. The resultant solution is enriched with fermentable sugars and nitrogenous compounds, enabling direct microbial fermentation without the need for additional enzymes or nutrient supplements. By obviating the requirement for extensive pretreatment, this simplified approach reduces process complexity, minimizes energy consumption, and potentially lowers operational costs associated with bio-conversion. Fundamentally, the advantageous biomass composition of tobacco facilitates a milder conversion process, which can streamline biorefinery design and enhance the overall energy efficiency of biofuel production.

In comparison to other feedstocks, the autoclave approach’s efficiency is notable. Previous studies have also examined tobacco biomass conversion and ethanol yields. For example, Gong et al. (2023) found that tobacco stem waste yielded ~87 g/L of ethanol (about 0.29 g ethanol/g dry biomass) after a mild acid–alkali pretreatment and enzymatic hydrolysis (Gong et al., 2023). This corresponds to roughly 367 L of ethanol per ton of tobacco biomass, a level comparable to the yields obtained from conventional energy crops. By contrast, untreated lignocellulosic feedstocks like switchgrass typically require extensive pretreatment to achieve similar sugar release, with an estimated ~100 gallons (~380 L) of ethanol per dry ton as a benchmark (Haque and Epplin, 2010). Tobacco’s advantage lies in achieving high solubilization (>65%) with minimal processing, potentially translating to high fermentation yields with lower input requirements. Additionally, earlier assessments (Barla and Kumar, 2019) have highlighted tobacco’s substantial biomass productivity, suggesting that optimized cultivation could produce competitive ethanol outputs per hectare relative to other crops. Overall, integrating these independent findings strengthens the view that tobacco can attain ethanol yields per unit biomass on par with sorghum, switchgrass, or *Miscanthus*, while benefiting from simpler conversion techniques.

## Life cycle assessment

In order to assess the environmental advantages of biofuels derived from tobacco, Wang et al. (2024) conducted an extensive life cycle assessment (LCA) comparing ethanol produced from tobacco leaves to conventional ethanol derived from corn stover (Wang et al., 2024). The LCA findings are promising: if tobacco were cultivated on marginal or degraded lands globally, the crop could potentially produce approximately 573 billion gallons of ethanol annually. More significantly, tobacco-derived ethanol was estimated to result in approximately 76% lower greenhouse gas emissions and energy consumption compared to conventional lignocellulosic fuels. The production of bioethanol from tobacco leaves was also linked to a notable reduction in cumulative fossil energy demand. These enhancements are attributed to both the high fermentable yield of tobacco biomass and the elimination of energy-intensive pretreatment processes. The study underscores the potential of tobacco to achieve high biofuel productivity with considerable climate mitigation benefits. Importantly, these benefits are optimized when tobacco cultivation is confined to non-food, marginal lands, thereby avoiding competition with food crops and minimizing the impacts of indirect land-use change. Incorporating tobacco into land-use strategies could potentially establish tobacco ethanol as a viable low-carbon fuel alternative.

## Valorization of agricultural residues

The tobacco industry annually produces significant agricultural residues and waste streams beyond the tobacco leaves themselves. These include low-grade tobacco, processing remnants, and tobacco dust, which consist of stems, midribs, roots, rejected leaves, and leaf dust. These materials are frequently discarded or incinerated, creating environmental disposal challenges due to their high nicotine content (Banožić et al., 2020; Abdellah et al., 2025; Zhou et al., 2025). Although Wang et al.’s proof-of-concept concentrated on fermenting tobacco leaf hydrolysate, a comprehensive biorefinery approach would aim to utilize the entire spectrum of tobacco biomass. Future research should investigate methods for detoxifying nicotine-rich residues, either chemically or enzymatically, recovering residual nutrients such as nitrogen, and converting fibrous fractions into valuable co-products. For instance, tobacco stalks and roots could be transformed into biochar, used for process energy through combustion, or subjected to anaerobic digestion to produce biogas. By developing strategies for nicotine detoxification and residue conversion (Barla and Kumar, 2019; Dang et al., 2023, Dang et al., 2025; Manthos and Tsigkou, 2025; Ramontja et al., 2025), it may be feasible to establish an integrated, zero-waste tobacco biorefinery. This approach not only addresses waste disposal challenges but also enhances resource efficiency and generates additional revenue streams through co-products.

## Microbial compatibility and detoxification

A critical factor in utilizing tobacco-derived media for fermentation is the presence of alkaloids, particularly nicotine, which can inhibit microbial growth at elevated concentrations. Nicotine and its related compounds are indeed toxic to numerous microorganisms (Dang et al., 2023; Chen et al., 2025; Dang et al., 2025). In the experiments conducted by Wang et al., mild autoclave conditions resulted in low nicotine levels in the hydrolysate, and the fermentation microbes tested, e.g. *E. coli* and yeast, exhibited minimal inhibition (Wang et al., 2024). However, for industrial-scale applications, a more comprehensive assessment is necessary. A diverse range of microorganisms, including resilient bacteria, yeasts, and filamentous fungi, should be evaluated for their tolerance to tobacco hydrolysates, with potential adaptation or engineering for enhanced nicotine resistance. For example, microbial strains could be evolved or genetically engineered to degrade nicotine or withstand its effects (Dang et al., 2023; Li et al., 2024; Dang et al., 2025). Concurrently, the implementation of targeted detoxification steps in the process could further optimize fermentation performance. Enzymatic pathways naturally exist in nature that can degrade nicotine into non-toxic compounds. The integration of such enzymes, such as bacterial nicotine dehydrogenases, into bioprocesses could effectively eliminate inhibitory alkaloids prior to fermentation (Tararina et al., 2021; Chen et al., 2022; Dulchavsky et al., 2023). By utilizing both tolerant microbial strains and detoxification strategies, it is possible to mitigate the inhibitory effects of the natural toxins present in tobacco, thereby ensuring consistent and efficient biofuel production from tobacco biomass.

## Policy alignment and socioeconomic considerations

The successful repurposing of tobacco for bioenergy applications necessitates alignment with existing policy frameworks and consideration of socioeconomic factors. In numerous countries, tobacco remains a critical cash crop that supports the livelihoods of smallholder farmers. Concurrently, international health agreements, such as the World Health Organization’s Framework Convention on Tobacco Control (FCTC), advocate for the provision of economically viable alternative crops to tobacco farmers, as outlined in Article 17, to reduce tobacco supply (Nguenha et al., 2021; Prowse and Pérez Niño, 2022; Ngoma et al., 2024). Redirecting tobacco agriculture towards the production of biofuels and bioproducts could thus facilitate a “just transition”, enabling farmers to sustain their incomes by cultivating tobacco for non-smoking purposes, while simultaneously advancing public health objectives and climate goals. Government intervention will be essential in mitigating the risks associated with this transition. Policy measures, such as subsidies or tax incentives for the cultivation of energy crops on marginal lands, carbon credit schemes that incentivize low-carbon fuel production, and low-interest loans or grants for the development of biorefinery infrastructure can foster a conducive environment for tobacco bioenergy initiatives. Furthermore, agricultural extension programs and training will be necessary to assist tobacco farmers in adapting to new production practices that prioritize biomass over leaf quality for cigarette production. In conclusion, a coordinated policy approach can align public health, rural development, and climate mitigation objectives, transforming a historically problematic crop into a component of a sustainable economic solution.

## Agronomic and genetic optimization

To enhance biomass yield on suboptimal soils, it is imperative to optimize both tobacco cultivation practices and plant genetics for biorefinery feedstock production. From an agronomic perspective, the implementation of modern precision agriculture tools can significantly improve resource efficiency and crop yields. Techniques such as remote sensing and drone imaging facilitate variable-rate fertilization, while AI-driven irrigation scheduling ensures optimal water use, even on marginal lands (Getahun et al., 2024; Mgendi, 2024; Aijaz et al., 2025; Chen, 2025; Saha et al., 2025). These approaches enable site-specific management strategies that enhance biomass production while minimizing inputs such as water, fertilizers, and pesticides.

Concurrently, genetic advancements in tobacco can improve its viability as a bioenergy crop. Through breeding and biotechnological methods, researchers are developing tobacco varieties with desirable traits, including low nicotine content, increased sugar accumulation, and modified cell wall composition for more efficient processing. The application of CRISPR/Cas9 genome editing in tobacco has demonstrated potential, for example, by knocking out nicotine biosynthesis genes to produce nicotine-free plants and targeting photosynthesis-related genes like Rubisco to enhance carbon fixation efficiency (Schachtsiek and Stehle, 2019; Donovan et al., 2020; Burner et al., 2022; Jeong et al., 2024). Advanced gene-editing delivery systems, such as RNA virus vectors, have facilitated non-transgenic genome modifications in tobacco, enabling trait enhancements, including reduced nicotine content, without the incorporation of foreign DNA (Zhang et al., 2023; Xiang et al., 2024). Traditional transgenic methods have also demonstrated efficacy in augmenting tobacco’s biomass potential. For instance, the overexpression of specific cell wall biosynthesis genes has been shown to increase plant size and biomass yield (De Caroli et al., 2022), while the introduction of stress-tolerance genes has enabled tobacco to sustain growth in saline or nutrient-deficient soils (Zhai et al., 2023). By integrating these agronomic and genetic advancements, it is feasible to develop specialized tobacco cultivars optimized for biorefinery applications. These cultivars would exhibit high biomass yield, reduced levels of anti-nutritional factors such as nicotine, and enhanced suitability for conversion into fermentable sugars (Schachtsiek and Stehle, 2019; Burner et al., 2022; Jeong et al., 2024; Deng et al., 2025). The development and implementation of these improved varieties will be essential to fully harness tobacco’s potential as a bioenergy crop.

## Sustainable land-use and ecosystem services

A pivotal strategy to mitigate competition with food production and reduce emissions from indirect land-use change involves relocating tobacco cultivation to marginal or degraded lands (Wang et al., 2024; Liu et al., 2025). Tobacco, being relatively resilient, can thrive in suboptimal soil conditions (Wang et al., 2025). Utilizing such lands for energy crop production can alleviate the demand on prime agricultural land (Jinger et al., 2025). Nonetheless, the long-term sustainability of these systems necessitates meticulous land and ecosystem management (Cheng and Li, 2024). Monoculture tobacco plantations, particularly on fragile lands, may result in soil degradation, biodiversity loss, or strain on water resources if not properly managed. Integrating tobacco cultivation into mixed land-use systems offers a potential solution. For example, intercropping tobacco with nitrogen-fixing cover crops or implementing agroforestry practices—such as planting tobacco alongside trees or shrubs—can enhance soil structure and fertility, improve water retention, and create habitats for beneficial organisms. These practices also provide additional benefits, including soil stabilization (which reduces erosion), carbon sequestration in vegetation and soils, and diversified income streams for farmers through the production of timber, fruits, or supplementary crops cultivated alongside tobacco. In the implementation tobacco biorefineries, land-use planning should be informed by comprehensive environmental impact assessments and regional planning to identify optimal sites that enhance benefits while minimizing ecological damage. Rigorous monitoring of soil health, groundwater quality, and surrounding biodiversity is essential to ensure that the conversion marginal lands to tobacco cultivation yields a net positive environmental impact. By conceptualizing tobacco-based bioenergy farming as an integrated agro-ecosystem, it is feasible to sustain or even enhance ecosystem services while generating feedstock for biofuels.

## Techno-economic feasibility and scaling

Although the technical feasibility and environmental benefits of tobacco biorefineries appear promising, their commercial viability hinges on techno-economic feasibility. To attract investment and achieve scalability, the cost of producing biofuels from tobacco must be competitive with alternative feedstocks and fossil fuels. Ongoing research and development (R&D) through pilot-scale and demonstration projects is necessary to refine processes and reduce costs. For instance, pilot biorefineries could play a crucial role in optimizing the mass and energy balances of the autoclave hydrolysis and fermentation processes, as well as in identifying potential bottlenecks when scaling up reaction volumes. Engineering research should prioritize the design of reactors and equipment tailored specifically for tobacco hydrolysate fermentation, in addition to the integration of downstream separation and purification processed (for ethanol or other products) to enhance overall efficiency.

From an economic modeling perspective, a comprehensive techno-economic analysis (TEA) is essential. This analysis should incorporate realistic estimates of feedstock logistics, such as the costs associated with harvesting, transporting, and storing bulky tobacco biomass, as well as the potential revenues from co-products, including electricity generated from lignin combustion or the sale of biochemicals derived from residues. Furthermore, it should assess the impact of policy incentives, such as subsidies, carbon pricing, on profitability. Including these variables will facilitate the determination of the minimum selling price of tobacco-based biofuels and the identification of key cost drivers. Preliminary TEA findings indicate that policy support could be pivotal in bridging the gap to economic viability, particularly during the initial deployment phase. As technology advances, economies of scale and process improvements are anticipated to enhance the economic viability of tobacco biorefineries. In conclusion, establishing a robust business case through pilot projects and economic analysis is crucial for transitioning tobacco biorefineries from conceptualization to commercial.

## Conclusion and outlook

Historically cultivated primarily for cigarettes and other smoked products, tobacco is now poised for a sustainable transformation. The study conducted by Wang et al. offers a compelling proof-of-concept for reimagining tobacco as a valuable feedstock for low-carbon biofuel production, rather than as a controversial cash crop. By employing mild autoclave treatment and fermentation, the researchers demonstrated that tobacco leaves can be converted into ethanol with significantly reduced greenhouse gas emissions and energy consumption compared to traditional lignocellulosic fuels.

When benchmarked against other dedicated energy crops, tobacco’s performance appears competitive. Lab-scale trials indicate that tobacco leaf hydrolysate can yield several hundred liters of ethanol per ton of biomass, similar to outputs from switchgrass or Miscanthus under standard conversion efficiencies (Haque and Epplin, 2010; Gong et al., 2023). Considering biomass productivity, tobacco cultivation can produce on the order of 10–15 dry tons per hectare (or more with intensive management) (Berbeć and Matyka, 2020), translating to a potential ethanol production of a few thousand liters per hectare. This falls within the range of reported ethanol yields for sorghum, switchgrass, and Miscanthus, which span approximately 2,000–8,500 L/ha depending on species and growing conditions (Tang et al., 2018; Lin et al., 2023). For instance, high-yield energy sorghum hybrids in optimal settings can reach ~15,000 L/ha ethanol (Tang et al., 2018), and Miscanthus can exceed 8,000 L/ha in favorable climates (Lin et al., 2023), whereas switchgrass often achieves around 4,000–5,000 L/ha in practice. Notably, the technological readiness of tobacco biofuel is still low (current efforts are at lab proof-of-concept, ~TRL 3–4), whereas conversion of grasses like switchgrass and Miscanthus has advanced to pilot or demonstration projects (higher TRLs). Closing this gap will require scale-up and optimization, but tobacco offers a unique opportunity to repurpose a legacy crop for clean energy. These comparisons indicate that with further development, tobacco can be on par with, or even complementary to, conventional energy crops in a future bioeconomy.

While this research provides a foundational step, unlocking the full potential of tobacco in the bioeconomy will necessitate interdisciplinary collaboration and innovation, integrating advancements in plant science, microbial engineering, process design, agronomy, and policy. Future endeavors should focus on several key areas: the development of specialized low-nicotine, high-biomass tobacco varieties; the application of precision agriculture techniques to cultivate tobacco sustainably on marginal lands; and the establishment of supportive policies and economic incentives to promote adoption. If these components are effectively integrated, a tobacco-based biorefinery system could offer multiple co-benefits, including the production of renewable fuels and biochemicals, the creation of rural economic development opportunities, and enhanced environmental outcomes, such as reductions in greenhouse gas emissions and increased soil carbon sequestration. Ongoing research, encompassing larger pilot projects and comprehensive life-cycle assessments, is essential to resolve existing uncertainties and to facilitate the safe and sustainable scaling of this concept. Through meticulous implementation, tobacco, historically associated with adverse health and environmental impacts, could be repurposed as a valuable resource for clean energy and climate solutions, thereby demonstrating the remarkable adaptability of agriculture in addressing global challenges.

## Data Availability

The original contributions presented in the study are included in the article/supplementary material. Further inquiries can be directed to the corresponding author.
